# In Vitro Observations of the Interactions between *Pholiota carbonaria* and *Polytrichum commune* and Its Potential Environmental Relevance

**DOI:** 10.3390/life11060518

**Published:** 2021-06-03

**Authors:** Daniel B. Raudabaugh, Daniel G. Wells, Patrick B. Matheny, Karen W. Hughes, Malcolm Sargent, Teresa Iturriaga, Andrew N. Miller

**Affiliations:** 1 Illinois Natural History Survey, University of Illinois Urbana-Champaign, Champaign, IL 61820, USA; dgwells2@illinois.edu (D.G.W.); amiller7@illinois.edu (A.N.M.); 2 Department of Environmental Engineering, Duke University, Durham, NC 27708, USA; 3 Department of Molecular and Cellular Biology, University of Illinois Urbana-Champaign, Champaign, IL 61820, USA; 4 Department of Ecology and Evolutionary Biology, University of Tennessee, Knoxville, TN 37996, USA; pmatheny@utk.edu (P.B.M.); khughes@utk.edu (K.W.H.); 5 Department of Plant Biology, University of Illinois Urbana-Champaign, Champaign, IL 61820, USA; malcolms@life.illinois.edu; 6 School of Integrative Plant Science, Cornell University, Ithaca, NY 14850, USA; ti14@cornell.edu

**Keywords:** agaricales, endophytes, fungal–bryophyte ecology, pyrophilous fungi, wildfire

## Abstract

Wildfires play a critical role in maintaining biodiversity and shaping ecosystem structure in fire-prone regions, and successional patterns involving numerous plant and fungal species in post-fire events have been elucidated. Evidence is growing to support the idea that some post-fire fungi can form endophytic/endolichenic relationships with plants and lichens. However, no direct observations of fire-associated fungal–moss interactions have been visualized to date. Therefore, physical interactions between a post-fire fungus, *Pholiota carbonaria*, and a moss, *Polytrichum commune*, were visually examined under laboratory conditions. Fungal appressoria were visualized on germinating spores and living protonemata within two weeks of inoculation in most growth chambers. Appressoria were pigmented, reddish gold to braun, and with a penetration peg. Pigmented, reddish gold to braun fungal hyphae were associated with living tissue, and numerous mature rhizoids contained fungal hyphae at six months. Inter-rhizoidal hyphae were pigmented and reddish gold to braun, but no structures were visualized on mature gametophyte leaf or stem tissues. Based on our visual evidence and previous work, we provide additional support for *P. carbonaria* having multiple strategies in how it obtains nutrients from the environment, and provide the first visual documentation of these structures in vitro.

## 1. Introduction

Wildfires play a critical role in shaping ecosystems, maintaining biodiversity, and influencing ecological processes in fire-prone regions [1]. Early post-fire colonizers include fire-associated fungal and bryophyte species that often occur in close spatial proximity [2,3,4]. Early colonizers are believed to be important in ecosystem recovery through nutrient retention, binding soil particles, and serving as mycorrhizal inoculants [2,3]. Unfortunately, there is a knowledge gap in our understanding of post-fire early colonizer life strategies, particularly with respect to post-fire fungi.

Post-fire fungi are categorized into two main categories based on their response to fruiting in burnt habitats [5]: (1) fruiting occurs only after a fire, and (2) fruiting can occur at any time, but fruiting is enhanced after a fire. *Anthracobia* spp., *Ascobolus* spp., *Morchella exuberans* Clowez, Hugh Sm. and S. Sm., *Peziza echinospora* P. Karst., *Plicaria* spp., *Psathyrella pennata* (Fr.) A. Pearson and Dennis, *Pholiota carbonaria* (Fr.) Singer, *Pyronema* spp., and *Rhizina undulata* Fr. are some species that require fire to fruit. Fungal species with enhanced fruit body production after a fire include *Hygrocybe conica* (Schaeff.) P. Kumm., *Mycena galericulata* (Scop.) Gray, *Sphaerosporella* spp., and *Thelephora terrestris* Ehrh. [5].

Similar to post-fire fungi, there are species of mosses and liverworts that rapidly colonize burnt areas after a forest fire. They are often the first species to colonize burnt substrates, especially after intense fires, and their role at the beginning of succession should not be underestimated. These include, but are not limited to, *Bryum argenteum* Hedw., *B. sauteri* Bruch and Schimp., *Campylopus introflexus* (Hedw.) Brid., *Ceratodon purpureus* (Hedw.) Brid., *Funaria hygrometrica* Hedw., *Polytrichum juniperinum* Hedw., and the post-fire liverwort, *Marchantia polymorpha* L. [6,7]. Juvenile gametophytes of *B. argenteum, C. purpureus*, and *F. hygrometrica* typically appear around six weeks after a fire, whereas *M. polymorpha* typically appears after several months [8]. There is an apparent lack of knowledge concerning the colonization of bryophytes on burnt wood, which is an essential topic when planning forest restoration treatments that aim to increase biodiversity. In relation to *P. carbonaria*, basidiomata of this species start to appear about five months after a fire event and continue fruiting for at least 18 months [5], placing both fungal spores and maturing bryophytes in close temporal and spatial proximity.

In times between fire events, studies have indicated that some post-fire fungi remain in the soil as active saprobes [9] or dormant as spores or sclerotia [10,11]. Other post-fire fungi appear to associate as endophytes [12,13], mycorrhizae [14,15], or pathogens [16]. Recently, Matheny et al. 2018 [17] proposed the body snatchers hypothesis, suggesting that some post-fire fungi form endophytic and/or endolichenic relationships with bryophytes and lichens. In addition, Raudabaugh et al. [18] provided support for the body snatchers hypothesis based on published GenBank sequences [15,19,20,21] and culture-dependent and metagenomic culture-independent analyses. They detected many post-fire fungi from bryophyte, lichen, and club moss samples. Similarly, U’Ren et al. [19] stated that more than half of bryophyte and lichen endophytes within the Arizona fire-dominated forests belong to post-fire fungal taxa including *Anthracobia*, *Geopyxis*, and *Pyronema*, suggesting an important relationship between bryophytes and some post-fire fungi taxa.

*Pholiota carbonaria* (syn. *P. highlandensis*) is a member of the family Strophariaceae, order Agaricales, and is a well-known post-fire fungus [22]. It has a cosmopolitan distribution throughout the Americas, East Asia, and Europe [17]. This species has been inferred to form associations with vascular plant species (genera *Pinus* and *Taxus*), bryophytes, and lichens [17]. *Pholiota carbonaria* has been previously isolated into pure culture (as *P. highlandensis* [17]) from surface-sterilized *P. commune* Hedg. and identified as an endophyte from surface-sterilized tissues of *Atrichum angustatum*, Bryaceae sp., *Cinclidium* sp., *Climacium americanum*, *Leucobryum* sp., *Lobaria quercizans* Michx, *P. commune*, and *Thuidium* sp. [18], and as a saprobe [23]. Due to the ubiquitous range of *P. commune* and the post-fire fungus *P. carbonaria*, ease of growing both species in vitro, and solid evidence that these species interact in nature qualify this fungal–host combination as a suitable model to understand post-fire fungal–bryophyte interactions.

Therefore, although there is currently ample evidence to support that some pyrophilous fungi can associate with bryophytes, there is a lack of visual documentation as to the extent (structures and location) of these specific fungal–moss interactions. Consequently, this study aimed to visually document the physical interactions (structures produced) in vitro between a post-fire fungus, *P. carbonaria*, and the moss, *P. commune*, and discuss the environmental relevance of this interaction.

## 2. Materials and Methods

### 2.1. Isolation/Sampling of Fungi

**Figure 1:**
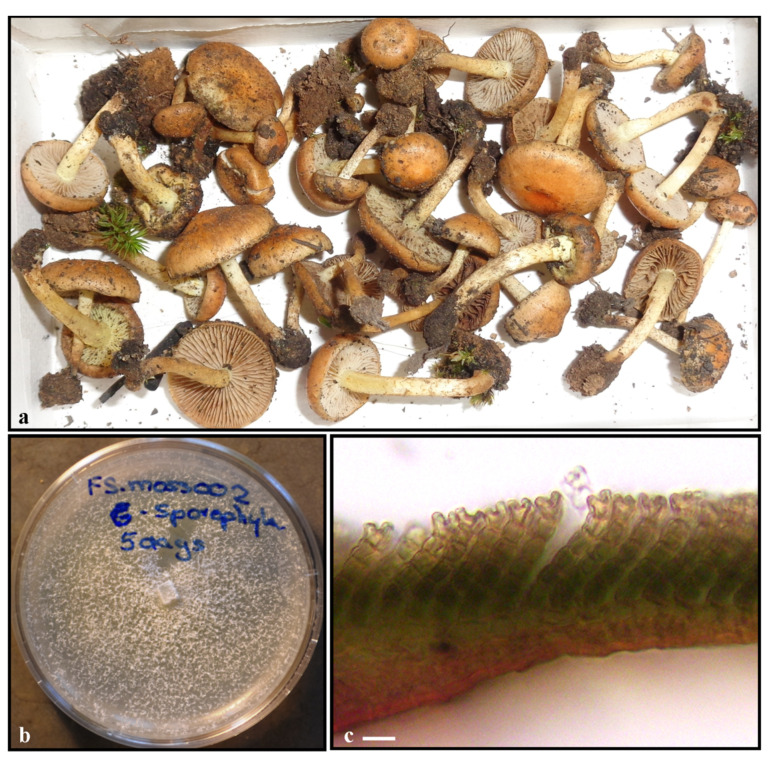
*Pholiota carbonaria* and *Polytrichum commune*. (**a**) *P. carbonaria* was collected in October 2017 from the Great Smoky Mountains National Park. Note several moss gametophytes in close proximity to the collected basidiomata. (**b**) *P. carbonaria* isolated from the moss *P. commune* in pure culture. (**c**) *P. commune* u-shaped terminal lamella cells of in vitro gametophyte. Scale bar = 20 µm.

We obtained *P. carbonaria* cultures from surface-sterilized moss tissues and from basidiomata collected from the Great Smoky Mountains National Park (Figure 1a,b). Tissues of several post-fire bryophytes were surface sterilized by washing in sterile distilled water followed by a 30 s immersion in 70% ethanol (EtOH), 30 s in 10% Clorox (sodium hypochlorite), and 30 s in 95% EtOH [13]. After surface sterilization, samples were placed on sterile paper towels to dry. Mosses were cut into small segments, including gametophyte and sporophyte tissues, where available and placed on malt extract agar (15 g Difco malt extract, 20 g Difco agar/liter) in 60 mm Petri dishes, sealed with Parafilm and incubated at room temperature. Cultures were examined every day for growth of fungal endophytes from moss fragments, and any observed endophytes were sub-cultured to new plates. To identify fungal endophytes, fungal cultures were sub-cultured to liquid potato dextrose broth and allowed to grow until sufficient tissue was available for DNA extraction. Cultured endophyte identities were confirmed through nucleotide analysis of the fungal internal transcribed spacer region (ITS) using the Basic Local Alignment Search Tool [24,25].

### 2.2. Growth Chamber Setup and Fungal Inoculation

**Figure 2:**
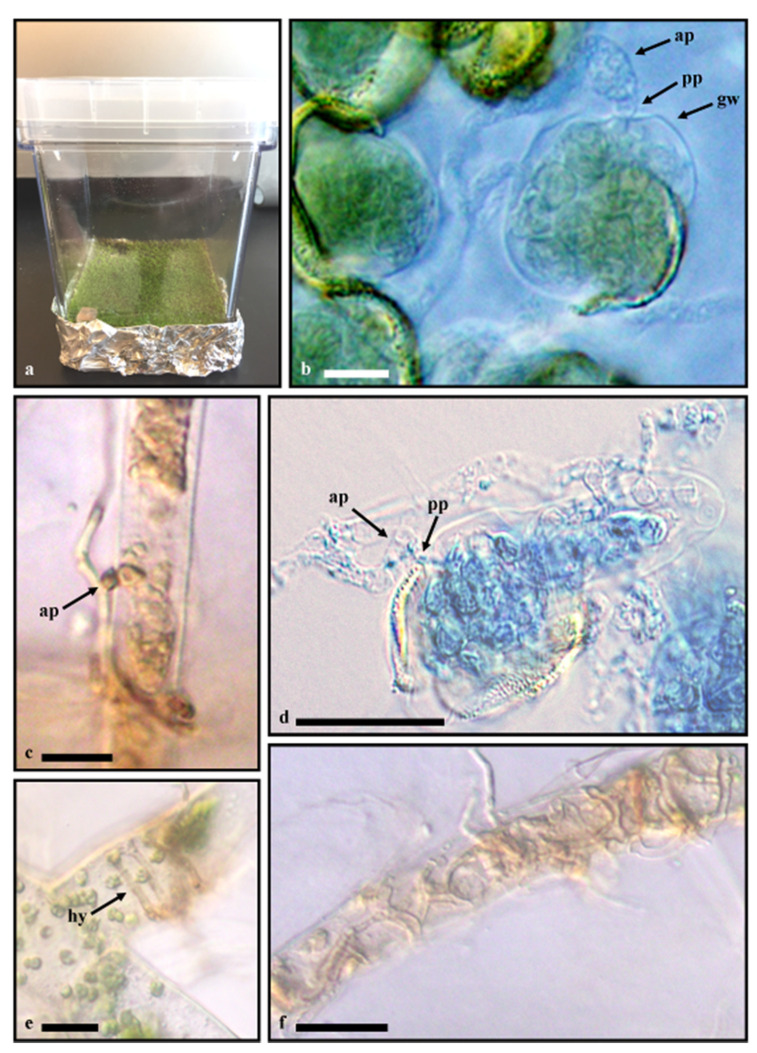
Associations of *Pholiota carbonaria* with *Polytrichum commune*. (**a**) Control growth chamber at three months; (**b**) fungal appressorium associating with germinating spore 14 days after inoculation; (**c**) appressorium attached to protonema at one month; (**d**) trypan blue stain showing continuous staining from appressorium into germinating spore, day 14 post-inoculation; (**e**) associated fungal hyphae with living tissue at two months; (**f**) mature rhizoids at six months. Scale bars **b**–**f** = 10 µm. ap = appressorium, pp = penetration peg, gw = germinating spore cell wall, and hy = fungal hyphae.

Modified BCD medium [26] was heated under constant stirring until agar was completely dissolved, and ~25 mL of media was added to 72 × 72 ×100 mm transparent square vessels (SPL Incu Tissue). This comprised the growth chamber (Figure 2a). Growth chambers were capped with lids, covered with aluminum foil, and autoclaved at standard temperature and pressure. After cooling, the bottom of each growth chamber was wrapped with aluminum foil 1–2 cm high (Figure 2a). Growth medium modifications consisted of the following: trace elements were added by using orchid fertilizer (Miracle Gro^®^ (Lowe’s, Mooresville, NC, USA) mixed per manufacturer instructions and one mL L^-1^ of this orchid fertilizer solution was added to the BCD medium before autoclaving. Ferrous sulfate was not added due to its inclusion within the orchid fertilizer.

*Polytrichum* moss spores were obtained from Carolina Biological Supply (item # 156661). Each growth chamber was inoculated with moss spores under a laminar flow hood. The outer surface of moss spore vials were wiped down with 100% ethanol before opening, *Polytrichum* spores were allowed to fall onto the medium surface in a fine layer, recapped, and set by a windowsill exposed to both partial direct and partial indirect light at 22 °C. After one week, growth chambers containing *Polytrichum* spores were visually inspected for bacterial and fungal contamination. Four growth chambers were randomly selected, and one corner was inoculated with one 4 × 4 mm agar plug containing actively growing *P. carbonaria* mycelia. This was repeated for 4 weeks to allow four different stages of *Polytrichum* development to be exposed to *P. carbonaria*. Negative controls consisted of growth chambers without *Polytrichum* spores and uninoculated *Polytrichum* spores. *Polytrichum*, species level identification, was determined at the end of the experiment to be *P. commune*, the most widely distributed *Polytrichum* species in the eastern United States [27], based on the u-shaped terminal lamella cells of the gametophyte (Figure 1c).

### 2.3. Microscopic Examination

Light microscopic evaluation was conducted using an Olympus BX51 microscope with differential interference contrast (DIC) equipped with an Olympus DP22 camera. Images were captured and measured using Olympus cellSens standard software version 1.18 (Olympus Corporation, Tokyo, Japan). Each week a small piece of medium with germinated *Polytrichum* spores was excised and examined for the presence of fungal–host interactions. Staining procedures using lactophenol cotton blue, trypan blue, and acidified trypan were used in an attempt to visualize the resulting structures [28] better. Color was determined using Kornerup and Wanscher [29].

### 2.4. Evaluation of Fungal Endophytic Association

Six-month-old *Polytrichum* gametophytes from inoculated and uninoculated control growth chambers were surface sterilized following a modified version of [30]. In short, moss gametophytes were transferred to plastic cassettes, submerged in distilled water with forceful agitation to remove external medium for 30 s, submerged for 10 s in 96% EtOH, 1 min in NaOCl solution (3% available Cl), and 10 s in 96% EtOH solution with a final rinse for 30 s in distilled water. All plastic cassettes were agitated throughout the entire surface sterilization process. Surface-sterilized gametophytes were placed onto malt extract agar (MEA) in 60 mm Petri plates, wrapped with Parafilm, incubated in the dark for 1–2 weeks, and periodically examined for fungal growth. Fungal colonies that grew out from the gametophytes were transferred to MEA plates.

### 2.5. Post-In Vitro Fungal DNA Extraction, Sequencing, and Identity

Fungal DNA was extracted by grinding fresh mycelium in 200 μL 0.5 M NaOH. The extract was centrifuged at 16,873× *g* for 2 min, and 5 μL of the resulting supernatant was added to 495 μL 100 mM Tris-HCl buffered with NaOH to pH 8.5–8.9 (Tris-HCl-DNA extraction solution [31]). PCR was completed on a Bio-Rad PTC 200 thermal cycler. The total reaction volume was 25 μL (12.5 μL GoTaq^®^ Green Master Mix (Promega, Madison, WI, USA), 1 μL of each 10 μM primer ITSIF and ITS4 [32,33], 3 μL of the Tris-HCl-DNA extraction solution and 7.5 μL DNA free water). Thermal cycle parameters consisted of an initial denaturation at 94 °C for 2 min, followed by 30 cycles of 94 °C for 30 s, 55 °C for 45 s, 72 °C for 1 min with a final extension step of 72 °C for 10 min. Gel electrophoresis (1% Tris-Borate- Ethylenediaminetetraacetic acid agarose gel stained with ethidium bromide) was used to verify the presence of a PCR product. Purification was completed using a Wizard^®^ SV Gel and PCR Clean-Up System (Promega). A BigDye^®^ Terminator 3.1 cycle sequencing kit (Applied Biosystems Inc., Foster City, CA, USA) was used to sequence the ITS in one direction using the ITS5 primer [32] on an Applied Biosystems 3730XL high-throughput capillary sequencer. Fungal isolate identity was confirmed through comparison to the pre-inoculation ITS sequence of *P. carbonaria* (GenBank number MT307288) [5].

## 3. Results

**Figure 3:**
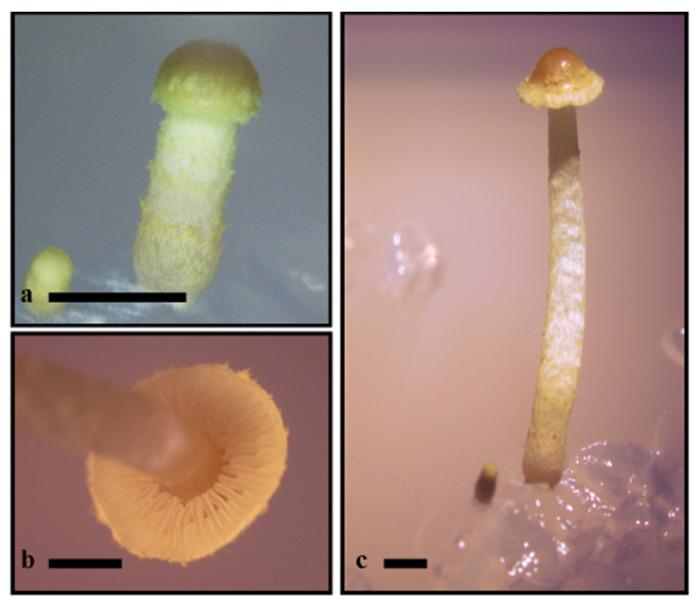
In vitro *Pholiota carbonaria* fruit body initials and mature basidiomata at two months post-inoculation. (**a**) Initial and developing basidiome. (**b**) Exposed lamellae. (**c**) Initial and basidiome. Scale bars: A–C = 5 mm.

Microscopic evaluation of *P. commune* germinating spores showed numerous hyphae of *P. carbonaria* in close proximity to germinating spores and protonemata. Hyphae of *P. carbonaria* had prominent clamp connections. No noticeable difference with inoculation time was perceived. Fungal appressoria were visualized on germinating spores and living protonemata (Figure 2b–d) within two weeks of inoculation in most growth chambers. Appressoria were pigmented, reddish gold to braun, 7.5–8.0 × 6.0–6.5 µm, with the penetration peg 1.5–2.0 × 1.5–1.8 µm (Figure 2b,c). Pigmented, reddish gold to braun fungal hyphae were associated with living tissue (Figure 2e), and numerous mature rhizoids contained fungal hyphae at six months (Figure 2f). Inter-rhizoidal hyphae were pigmented, reddish gold to braun, and 2–2.5 µm wide (Figure 2f). No structures were visualized on mature gametophyte leaf or stem tissues. *Pholiota carbonaria* was isolated from surface-sterilized six-month-old gametophytes, and the nBLAST analysis of the ITS rDNA region of the isolate was virtually identical (one base pair difference) original inoculants. No fungi were observed within negative controls and *P. carbonaria* was not isolated from uninoculated spore controls. In two months, several fruit body initials and mature *P. carbonaria* basidiomata were produced with viable basidiospores capable of generating new cultures (Figure 3).

## 4. Discussion

We have documented that *P. carbonaria* can form structures associated with living germinating moss spores (Figure 2b,d) and protonema via appressoria (Figure 2c) and penetration pegs (Figure 2b,d) and can colonize mature rhizoids of *P. commune* in vitro (Figure 2f) but with unapparent and asymptomatic infection. This association is believed to be endophytic based on definitions outlined by Wilson [34] which suggested that all endophytes are either commensals or parasites since they derive all their nutrients from the host but cause unapparent and asymptomatic infection and have at least some of their life cycle inside plant tissues. This is in contrast to pathogens that cause apparent and symptomatic infections. We also documented the production of mature basidiomata of *P. carbonaria* in vitro (Figure 3a–c) for the first time. Since numerous cultures of *P. carbonaria* held at the University of Tennessee did not fruit in culture, even after extended time, it is tempting to suggest that fruiting may be affected by the presence of moss gametophytes but further investigation is needed. It is possible that the colonization of young gametophyte structures and mature rhizoids may represent an opportunistic in vitro association and not necessarily an in vivo endophytic relationship. However, taking into account all the data collected thus far by various authors, we believe there is ample support to believe the fungal–host interactions documented here represent what can occur in nature.

*Pholiota carbonaria* has been previously isolated from many bryophyte species (discussed below) and was found in a large number (42%, 16 of 38 samples) of surface-sterilized non-necrotic moss and lichen tissues [18]. Although fungal–bryophyte associations were thought to be uncommon, this is now believed to be a misconception. Because of the unique morphological and physiological makeup of bryophytes, Davey and Currah [35] suggest further study is needed to determine if fungal–moss interactions are similar and as common as fungal–vascular plant interactions.

The historical understanding of fungal–moss associations appears to vary within the literature. Pressel et al. [36] reported that fungal–moss symbiotic relationships have not been demonstrated and that most fungal structures are confined to dead or senescing host cells. By contrast, Zhang and Guo [37] investigated arbuscular mycorrhizal structures in mosses and found vesicles, intracellular hyphal coils, and intercellular aseptate hyphae in living stems and leaves but not rhizoids of mosses. Zhang and Guo [37] also concluded that the presence of these structures was not evidence for a mutualistic symbiosis; however, there are other associations beyond mutualistic symbiosis that may represent a biotrophic association. Interestingly, six genera within the Ascomycota family Pyronemataceae, of which some species are found in burned areas, are obligate symbionts with bryophytes [20,38]. They produce hyphae that are attached to living rhizoids via appressoria with intracellular haustoria [39]. Previous research has suggested that these fungi are likely to obtain some nutrients from their host [20,40].

Species within the Basidiomycota have also demonstrated the ability to form associations with bryophytes, including parasitic/pathogenic [41], and saprobic [42] relationships. Although parasitic/pathogenic interactions occur, the funal–host interactions vary [35]. Appressoria are common in vascular plant–fungal biotrophic interactions [43], but although not absent, they are less common in bryophyte pathogenic fungi [35], which form penetration pegs and use extracellular enzymes. Redhead [41] investigated the interactions between *Galerina paludosa* (Fr.) Kühner and *Sphagnum capillifolium* (Ehrh.) Hedw. (formally, *Sphagnum capillaceum* (Weiss) Schrank). Moreover, it was concluded that they form a parasitic relationship. Redhead [41] visualized peg-like haustoria at day 14, and by one month, he noted infection of cells of the protonema, rhizoids, and prothallus, in addition to hyphae growing through dead cells [41]. Further evidence of biotrophic interactions between fungi and bryophytes were completed by Korotkin et al. [44]. They found stable isotopic evidence providing direct evidence for numerous biotrophic interactions between fungi in the order Hymenochaetales and bryophytes.

Our study visualized what appeared to be appressoria with penetration pegs (Figure 2b–d) occurring on germinating spores and young gametophytes, suggesting an initial parasitic relationship similar to that seen by [41] but there was no evidence of necrosis. Davey and Currah [35] suggested that since *G. paludosa* infects only the initial filaments from germinating spores, this relationship may be a pioneering life-history strategy or that *G. paludosa* attacks immature gametophytes before the host can mount a resistance. Based on the current visual evidence and previous work, *P. carbonaria* has plasticity in its nutritional relationship [34] with *P. commune*, which is not uncommon among fungal species [45]. The time line of and structures visualized in our study are similar to what has been reported for *G. paludosa* but with unapparent and asymptomatic infection. Furthermore, *P. carbonaria* is also likely saprobic within the dead tissue layers through the invasion of dead rhizoids over time and is capable of growing independently in vitro on malt extract and potato dextrose [5,18]. Although no infection in mature leaf and stem tissues was visually evident in this study, *P. carbonaria* was previously isolated from surface-sterilized non-necrotic leaf and stem tissues from several moss species.

### Environmental Relevance

Fungal–moss associations may be common within the genus *Pholiota*. Chen et al. [46] detected two unidentified species of *Pholiota* from eastern North Carolina from culture-dependent studies of the moss *Dicranum scopiarum* Hedw. ITS sequence data of these *Pholiota* isolates indicated that they were *P. castanea*, another post-fire *Pholiota* [17] and *P. spumosa*, a non-post-fire species of *Pholiota*. Raudabaugh et al. [18] isolated several cultures of *P. carbonaria* from surface-sterilized moss samples collected from recently burned sites in the Great Smoky Mountains National Park. The moss species from which these isolates were obtained included one fire-response moss, *Funaria hygrometrica* Hedw., and several common moss species such as *Ceratodon purpureus* (Hedw.) Brid., *Ditrichum pallidum* (Hedw.) Hampe, and *P. commune*. Another species, *Pholiota castanea*, was also identified from *Atrichum angustatum* (Brid.) Bruch and Schimp. [18]. *Pholiota peleae* E. Horak and Desjardin was described from Hawaii, and although not a post-fire *Pholiota* species, this species was initially described from bryophyte covered bark of living or dead *Metrosideros polymorpha* Gaudich [43] in addition to trunks of *Cibotium glaucum* (Sm.) Hook. and Arn. (a tree fern). *Pholiota henningsii* (Bres.) P.D. Orton is considered sphagnicolous, inhabiting or growing on *Sphagnum*, and is found rarely in several European countries [42]. It is believed that this *Pholiota* species likely break down lignin-like polymers and is not considered to parasitize *Sphagnum* species [42]. Finally, although not a *Pholiota*–bryophyte interaction, several *Pholiota* species were found in biological soil crusts (composed of fungi, algae, and cyanobacteria) in glacier forelands in Southern Norway [47], suggesting that *Pholiota* species have many life-history strategies that allow them to occupy a wide variety of poor nutrient habitats.

These previous studies and our current visual confirmation provide additional evidence that some fire-associated and non-post-fire species of *Pholiota* associate with mosses in nature. Notably, the broad range of moss species that *P. carbonaria* and other fire-associated fungi have been identified with in nature, along with the current in vitro observations of this association, suggest that the fungi–moss interaction may provide a more favorable environment (consistent source of nutrients, nitrogen, leachate, and moisture) than the surrounding soil. Depending on the fire intensity, burnt soil is low in some nutrients and has altered soil physical properties [48], including reducing the shallow organic matter layer, nitrogen, and water holding capacity. Interestingly, De Las Heras et al. [3] demonstrated that total nitrogen levels under bryophyte turfs were higher than the surrounding soil, which may be beneficial for fungal growth if soil nitrogen levels are insufficient post-fire event. In addition, the ability to colonize both fire-associated mosses and later-stage successional moss species would provide the same long-term life strategy.

In summary, based on visual evidence and previous work, we show that *P. carbonaria* can form appressoria with penetration pegs, and invade mature rhizoid in vitro on *P. commune* and suggest that this post-fire species has multiple strategies in how it obtains nutrients from the environment. Future analyses of stable carbon and nitrogen signatures would be of value in fully understanding the nutritional relationship, electron microscopy visualization would be beneficial in further refining the interactions between *P. carbonaria* and *P. commune*, and a long-term study is needed to determine the temporal stability of this association. Although additional research is needed to understand this relationship fully, this study suggests that early post-fire colonizers may have more interconnected life histories than previously thought, which is essential when considering the contribution of post-fire fungi and bryophytes to post-forest fire system recovery.

## Data Availability

Data are available upon request.
